# Multidrug-resistant gram-negative bacteria in Spanish ICU patients: clinical and microbiological characterization (MURAN-UCI Project)

**DOI:** 10.1128/spectrum.02987-25

**Published:** 2025-12-26

**Authors:** E. Ramirez de Arellano, C. López-Causapé, M. Delgado-Valverde, F. J. Arroyo Muñoz, E. Alemparte-Pardavila, J. Arca-Suárez, I. Ayestarán, J. Calvo Montes, J. Cañada-Garcia, S. Garcia-Cobos, S. García-Fernández, D. Gijón Cordero, J. J. González-López, A. Mir-Cros, X. Nuvials, M. Pérez-Vázquez, A. Pomares-de la Peña, M. Pampín-Garcia, C. Riazzo, J. Rodríguez-Gómez, E. Rojo-Molinero, P. Ruiz-Garbajosa, C. Soriano, B. Suberviola Cañas, B. Taltavull, J. Garnacho-Montero, A. Oliver Palomo, J. Oteo-Iglesias, On behalf of the MURAN-UCI Spanish group

**Affiliations:** 1 Centro Nacional de Microbiología, Instituto de Salud Carlos III 38176, Madrid, Spain; 2 CIBER Enfermedades Infecciosas (CIBERINFEC), Madrid, Spain; 3 Servicio de Microbiología, Hospital Universitario Son Espases, IdISBa, Palma de Mallorca, Spain; 4 Hospital Universitario Virgen Macarena/CSIC/Universidad de Sevilla, UGC Enfermedades Infecciosas y Microbiología, Instituto de Biomedicina de Sevilla (IBiS) 16582, Seville, Spain; 5 Unidad Clínica de Cuidados Intensivos, Hospital Universitario Virgen Macarena 16582, Sevilla, Spain; 6 Servicio de Medicina Intensiva, Complejo Hospitalario Universitario A Coruña 16811, A Coruña, Spain; 7 Servicio de Microbiología & Instituto de Investigación Biomédica A Coruña (INIBIC), Complejo Hospitalario Universitario A Coruña 548755 , A Coruña, Spain; 8 Servicio de Medicina Intensiva, Hospital Universitario Son Espases 375118, Palma de Mallorca, Spain; 9 Servicio de Microbiología, Hospital Universitario Marqués de Valdecilla, IDIVAL, Santander, Spain; 10 Servicio de Microbiología, Hospital Universitario Ramón y Cajal, Instituto Ramón y Cajal de Investigación Sanitaria (IRYCIS) 16507, Madrid, Spain; 11 Servicio de Microbiología, Hospital Universitario Vall d’Hebron 571818 , Barcelona, Spain; 12 Servicio de Medicina Intensiva, Hospital Universitario Vall d’Hebron 537649 , Barcelona, Spain; 13 Unidad de Gestión Clínica de Microbiología. Instituto Maimónides de Investigación de Córdoba (IMIBIC), Hospital Universitario Reina Sofía de Córdoba 16501, Córdoba, Spain; 14 Servicio de Medicina Intensiva, Hospital Universitario Reina Sofía de Córdoba 16501, Córdoba, Spain; 15 Servicio de Medicina Intensiva, Hospital Universitario Ramón y Cajal 16507, Madrid, Spain; 16 Servicio de Medicina Intensiva, Hospital Universitario Marqués de Valdecilla, IDIVAL 542557 , Santander, Spain; 17 Unidad Clínica de Cuidados Intensivos, Hospital Universitario Virgen de Rocío, Sevilla, Spain; Johns Hopkins University, Baltimore, Maryland, USA

**Keywords:** multi-resistance, gram-negative bacteria, ICUs, colonizations, infections

## Abstract

Multidrug-resistant gram-negative bacteria (MDR-GNB) are one of the main challenges in intensive care units (ICUs), mainly extended-spectrum β-lactamase-producing *Klebsiella pneumoniae* (EKP), carbapenemase-producing Enterobacterales, carbapenem-resistant (CR) *Pseudomonas aeruginosa,* and CR *Acinetobacter* spp. The objectives were to determine the prevalence and incidence of colonization/infection by these bacteria in Spanish ICUs, their antimicrobial susceptibility, resistome, and phylogeny. All patients admitted to ICUs of seven Spanish hospitals were evaluated (15 February to 30 March 2023). Colonization was determined in rectal and oropharyngeal swabs upon admission and the first and second weeks after admission. Infections were studied for up to 60 days from admission. Antimicrobial susceptibility was determined by broth microdilution. Whole-genome sequencing was performed to analyze the resistome and phylogeny (core-genome Multi-Locus Sequence-Typing). In total, 767 patients were studied. The prevalence of MDR-GNB colonization on admission was 2.9% (23/767), and EKP was the most prevalent bacterium. The incidence of acquired colonization was 1.3 (22/1,694) cases per 100 ICU-admitted patients-day, with colonization by CR *P. aeruginosa* being the most frequent (*n* = 14/22). Twenty-one patients had an infection (prevalence: 2.7%), mainly CR *P. aeruginosa* (12/21). The major resistance mechanisms and clones detected were some of the most frequent circulating clones in Spain: EKP-ST15/*bla*_VIM-1+OXA-48_, EKP-ST307/*bla*_CTXM-15_, and CR *P. aeruginosa* ST175/OprD porin deficiency. The antibiotics with the greatest activity against Enterobacterales (*n* = 26) were colistin, cefiderocol, and meropenem-vaborbactam (100%); while in CR *P. aeruginosa* (*n* = 20), they were colistin, cefiderocol, and imipenem-relebactam (95%–100%). The prevalence/incidence of colonization/infection by MDR-GNB in the ICUs of this study was low, but with important variations between hospitals. CR *P. aeruginosa* was the most common bacterium identified.

**IMPORTANCE:**

This comprehensive study integrates incidence/prevalence, clinical, microbiological, and genomic data to provide an up-to-date overview of multidrug-resistant gram-negative bacteria in Spanish intensive care units (ICUs). Its main strengths lie in its multidisciplinary approach and in the involvement of seven ICUs from major university hospitals across different regions in Spain. We believe that monitoring resistance epidemiology at the ICU level—especially through molecular surveillance of resistance mechanisms and clonal dissemination—is essential for optimizing antimicrobial use and guiding stewardship and infection control programs. Notably, carbapenem-resistant *Pseudomonas aeruginosa* was the most frequently identified organism in both colonization (first/second week) and infection cases, highlighting the need for its inclusion in routine hospital colonization screening.

## INTRODUCTION

Antimicrobial-resistant bacteria (ARB) are a major threat to human health worldwide (1). The horizontal transfer of resistance genes by mobile genetic elements, the spread of successful multidrug-resistant high-risk clones, and the presence of colonized patients are among the main factors that determine its spread (2–6). Screening for the detection of patients colonized by ARB has a double purpose: (i) epidemiological, by establishing early control measures, and (ii) clinical, by improving the empirical therapy of infections in previously colonized patients (7–10). Patients admitted to the intensive care units (ICUs) are more vulnerable to invasive infections by ARB due to the accumulation of predisposing factors and the complexity of nosocomial infections (11–13).

Although there are multiple species of ARBs that can cause infections and outbreaks, multidrug-resistant gram-negative bacteria (MDR-GNB) are the main challenge in ICU infections around the world (12, 13), although with a great geographic variability (11–13). WHO establishes the carbapenemase-producing Enterobacterales (CPE) and the extended-spectrum β-lactamases (ESBL)-producing Enterobacterales, and carbapenem-resistant (CR) *P. aeruginosa and Acinetobacter* spp. as critical or high priority ARBs (14).

In ICUs, admission and weekly universal screenings help to detect new MDR-GNB carriers. The lack or delay in MDR-GNB carrier detection can lead to an increased risk of cross-transmission (15). A study carried out in 2014 in 11 Spanish ICUs detected an average incidence of ICU-acquired CPE colonization of 1.6%, with 4% of them having an incidence greater than 3% (16). Several studies have observed that carriage of CPE places patients at risk of antibiotic-resistant infection (8), increasing length of hospital stay (17), and mortality (10).

The integration of new technologies, such as whole-genome sequencing (WGS), allows a comprehensive knowledge of the phylogenetic, resistome, virulome, and mobile genetic elements of ARB (2, 6, 18). The integration of WGS in ARB surveillance and diagnosis is a priority of the European Center for Disease Prevention and Control (ECDC) (19).

A broad and multidisciplinary approach to this problem will facilitate the translation of the results to the control measures and clinical practice, to reduce the impact of multidrug resistance in ICUs. So, this is a comprehensive study aimed to determine the general impact of MDR-GNB in Spanish ICUs, analyzing prevalence, incidence, and clinical conditions of colonization/infection by these MDR-GNB at admission to the ICU, the rate of subsequent infections by these bacteria in the 60-day follow-up, and the molecular characterization by WGS of the involved bacteria.

## MATERIALS AND METHODS

### Design, patients, participating hospitals, and isolates

All patients admitted to the ICUs of seven acute-care Spanish hospitals during 15th February to 30th March 2023, were included in this prospective, observational, and multicenter study. The participant hospitals were in seven different Spanish provinces and six autonomous communities (Table 1).

**TABLE 1:** Characteristics and participation of the university hospitals and ICU departments

Hospitals	Province (AC)	N° total beds	Transplant program^*a*^	N° ICU beds	ICU admissions/year^*b*^	Average length of ICU patients stay (days)^*b*^
H1	Sevilla (Andalucía)	700	No	30	1,354	6.75
H2	Palma de Mallorca (Islas Baleares)	735	Yes	36	1,408	8.3
H3	Córdoba (Andalucía)	1,400	Yes	40	1,400	4.5
H4	Santander (Cantabria)	907	Yes	36	1,769	5.8
H5	Barcelona (Cataluña)	1,100	Yes	36	1,700	8
H6	Madrid (Madrid)	900	Yes	36	1,200	7.7
H7	A Coruña (Galicia)	1,423	Yes	38	1,887	5.9

^*a*^ Hospital with solid organ and/or hematological transplant program (Yes/No).

^*b*^ 2023 data. AC: autonomous community.

According to the prioritization established by the WHO (14), the MDR-GNB undergoing surveillance in this study were de CPE, extended-spectrum β-lactamase-producing *Klebsiella pneumoniae* (EKP), CR *P. aeruginosa,* and *Acinetobacter* spp. Carrier status was tested in rectal and oropharyngeal swabs at the time of ICU admission, the first week (±2 days) and the second week (±days) of admission. Rectal and oropharyngeal swabs were cultured on selective commercial chromogenic agar (CHROMID CARBA SMART and ESBL [BioMérieux, France]) for the detection of CPE- and ESBL-producing Enterobacterales, and MacConkey agar with 2 mg/L of meropenem for the detection of CR *P. aeruginosa and Acinetobacter* spp. After 24 h of incubation at 37°C, all isolates growing on selective plates were identified at the species level by MALT-TOF. Additionally, infections by these MDR-GNB, at the time of ICU admission and during the stay up to 60 days, were also registered, and the strains involved were subjected to microbiological analysis along with those from colonization.

### Prevalence and incidence of colonization by CPE, EKP, and CR *P. aeruginosa* and *Acinetobacter* spp.

The prevalence of colonization by CPE, EKP*,* and CR *P. aeruginosa* and *Acinetobacter* spp. at the time of ICU admission was calculated as the number of colonized patients/total number of ICU-admitted patients during the study period. The incidence of colonization by these bacteria after 1 and 2 weeks of ICU admission was calculated as colonized patients per 100 patients-days ([number of patients colonized during the first/second week of ICU stay/total of ICU patients-days in first/second week] × 100).

Infections caused by bacteria under surveillance at the time of ICU admission and during the patients’ stay up to 60 days were also recorded. The MDR-GNB infections were diagnosed by the local clinical researchers who treated the patients based on signs or symptoms. The site of infection was also decided by the local clinical researchers, considering the sample where the microorganism was isolated, together with focal signs and symptoms, biological markers, and imaging tests.

### Clinical features

Clinical data were available for 653 patients (85.1%; 653/767): age, sex, type of admission, Charlson comorbidity index, and severity of the illness evaluated by the Acute Physiology and Chronic Health Evaluation (APACHE) II score, considering the worst data point for the first 24 h in the ICU, and prior antimicrobial therapy (at least for 2 days in the last 30 days) (Table 2). In those patients colonized or infected by MDR-GNB monitored during the study period, the following variables were registered: pre-existing comorbidities (as defined by APACHE II score [20]), travel abroad in the previous year, antimicrobial therapy in the previous 30 days, need for mechanical ventilation, and use of renal replacement therapy in the ICU. All admitted patients were followed up until death or hospital discharge for a maximum of 60 days. A standardized form was developed using OpenClinica’s electronic data capture platform (https://www.openclinica.com/solutions/electronic-data-capture-edc/) to collect all data prospectively.

**TABLE 2:** Clinical data of 653 ICU patients from seven Spanish university hospitals, 15 February to 30 March of 2023^*a*^

	Total cohort (*n* = 653)	Colonized patients (*n* = 29)	Infected patients (*n* = 21)	Non colonized/infected patients (*n* = 603)	*P* value
Age (years)^*b*^	63 (53–71)	66 (55–77)	62 (58–77)	66 (56–74)	0.94
Gender (male)^*c*^	61.9%	62.1	85.7	60.8%	0.06
Charlson Comorbidity Index^*b*^	2 (1–4)	3 (2–6)	3 (2–6)	3 (1–4)	0.28
APACHE II score^*b*^	14 (10–21)	19 (14–24)	23 (19.5–27.75)	14 (10–19)	0.0001
Previous hospital stay (days)^*b*^	0 (0–2)	1 (0–10)	6 (0–20)	0.5 (0–1.75)	0.006

^*a*^ Main clinical data collected.

^*b*^ For the analysis of quantitative variables, the Kruskal-Wallis test was used.

^*c*^ For the analysis of qualitative variables, the exact Fisher test was used.

### Antibiotic susceptibility testing

Antibiotic susceptibility testing was determined by broth microdilution (21) using Sensititre Gram Negative panels DKMGN and MDRGNX2F for Enterobacterales, and FRCNRP2 for *P. aeruginosa and Acinetobacter* spp.; these panels also include colistin (Thermo Fisher, Waltham, MA, USA), according to EUCAST 2024 (v14.0) guidelines (22). Additionally, the susceptibility to cefiderocol, and for *P. aeruginosa* imipenem/relebactam too, was tested by the broth microdilution method following the procedure recommended by EUCAST (23).

### WGS and read assembly

Genomic library preparation and sequence analysis were carried out as previously described (6). For Enterobacterales, the quality of the reads was assessed using FASTQC (version 0.11.9), followed by *de novo* assembly using Unicycler (version 0.4.8) (24). For *P. aeruginosa* and *Acinetobacter* spp., raw reads were trimmed with Trimmomatic v0.39 and *de novo* assembled with SPAdes v3.15.5 using the -careful option.

Raw sequence data were deposited in the European Nucleotide Archive: PRJEB78527 (Enterobacterales) and PRJEB88879 (*P. aeruginosa* and *Acinetobacter* spp.).

### Phylogenetic analyses

For *K. pneumoniae*, Ridom SeqSphere+ (version 8.3.1; Ridom, Münsten, Germany) was used to perform a core-genome Multi-Locus Sequence-Typing analysis (cgMLST) using built-in schemes containing 2,538 core genes. ARIBA (version 2.14.6) (25) was used to determine sequence types (STs) in accordance with the Institute Pasteur scheme for *K. pneumoniae* and the *Public databases for molecular typing and microbial genome diversity* schemes (PubMLST) (https://pubmlst.org/organisms/enterobacterales) for other enterobacteria.

For *E. hormaechei* and *K. oxytoca, ad hoc* cgMLST schemes were created using the MLST+ target definer with the default parameters and a reference sequence: *E. hormaechei* (accession no NC_021046) and *K. oxytoca* KONIH1 (accession no NZ_CP008788.1). A total of 24 and 13 NCBI RefSeq genomes for *E. hormaechei* and *K. oxytoca*, respectively, were used as query genomes to validate in a pairwise comparison using the BLAST program. The final cgMLST schemes consisted of 2,123 target genes for *E. hormaechei* and 3,201 for *K. oxytoca*.

For *P. aeruginosa* and *Acinetobacter* spp., the *novo* assemblies were used to infer ST with MLST v2.23.0 (https://github.com/tseemann/mlst) and using the pubMLST databases. A cgMLST was performed for *P. aeruginosa* using the open-source algorithm chewBBACCA (26), and a minimum spanning tree based on 4,201 genes was constructed with Grape Tree (27).

Clusters were described applying a threshold of ≤4 alleles of difference for *K. pneumoniae* and *E. hormaecheii* and ≤6 alleles for *P. aeruginosa* (28, 29).

### Analysis of genetic determinants of antibiotic resistance

Acquired antibiotic resistance genes (ARGs) in Enterobacterales were analyzed by ARIBA (version 2.14.6) (25) using the CARD database (https://card.mcmaster.ca; accessed 13/04/2024) and ResFinder (CGE server, https://bitbucket.org/genomicepidemiology/resfinder_db/src/master/, accessed on April 2024) with ID thresholds of 100% for β-lactamase variants and 98% for other resistance genes.

For *P. aeruginosa,* the *de novo* assemblies were used to explore the presence of acquired resistance determinants as well as the structural integrity of the OprD porin as previously described (30). In addition, a variant calling analysis was performed to study the mutational resistome. For this purpose, the Snippy software v4.6.0 (https://github.com/tseemann/snippy) was used with the *P. aeruginosa* PAO1 genome (NC_002516.2) as a reference. Single-nucleotide variants and short insertions and deletions (InDels) found in a set of 57 genes involved in antibiotic resistance were extracted and filtered using a list of substitutions previously classified as natural polymorphisms (31). Gene absence was also investigated using the SeqMonk visualization tool (https://www.bioinformatics.babraham.ac.uk/projects/seqmonk/).

### Statistical analysis

The Kolmogorov-Smirnov test was applied to quantitative variables to determine whether they had a normal distribution. Quantitative variables are expressed as median values (percentile 25–percentile 75) because not all of them were normally distributed. Qualitative variables are expressed as percentages. The analysis was performed using Fisher’s exact test for qualitative variables and the Kruskal-Wallis test for quantitative variables.

## RESULTS

### Patients, isolates, and clinical features

**Fig 1:**
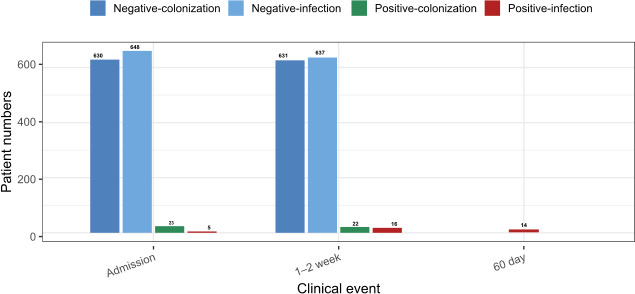
Timeline of MDR-GNB colonized (*n* = 29), infected patients (21; 5 infected and 16 colonized/infected), and non-colonized/infected patients (*n* = 603) at ICU admission, stay, and up to 60 days of stay for infections detected during the period of study.

In total, 252 ICU beds were surveyed. Table 1 shows the main characteristics of hospitals and ICU departments participating. A total of 767 patients were included; the screening strategy implemented in this study is routinely practiced in the ICUs of the participating hospitals, which facilitated the recruitment of all patients admitted during the study period. Of them, 281 (36.6%) and 121 (15.8%) remained in the ICU for at least 1 and 2 weeks, respectively. Fifty (6.5%) patients were colonized (*n* = 29) or infected (*n* = 21) by the MDR-GNB subject to surveillance during the study period, including cases from the time of admission to the second week of ICU stay for colonizations, or to up to 60 days of stay for infections (Fig. 1). In three patients, two or more bacterial species/resistance indicators were collected. In 19 patients, more than one isolate of the same bacterial species/resistance indicator was studied; 10 of them because both isolates from colonization and infection were included, and in 12 patients because isolates from different sample types or pick-up time were also included.

Ccal data were collected from 653 of the 767 (85.1%) patients (Table 2); in the remaining 14.9%, this information could not be recovered. Patients colonized (*n* = 29) and infected (*n* = 21) by the MDR-GNB subject to surveillance had significantly longer stay in the hospital ward before being admitted to the ICU and a more severe condition assessed by the APACHE II score (Table 2). Thirty of the 50 (60%) patients colonized or infected by MDR-GNB presented any of the recorded underlying diseases. The most frequently pre-existing comorbidities were chronic renal insufficiency (*n* = 9), cirrhosis (*n* = 7), and chronic cardiac insufficiency (*n* = 7). Thirty patients had received antimicrobial therapy in the previous 30 days, 19 were on mechanical ventilation at any time during ICU admission, and 9 required renal replacement therapy. Three patients reported traveling abroad (two European countries and one to South America).

### Prevalence and incidence of colonization by MDR-GNB

On admittance, the prevalence of MDR-GNB colonization among carriers was 2.9% (23 of 767 patients) (interhospital range: 0.5%–10.3%) (Table 3). The colonizing bacteria were as follows: 11 EKP (1.4%), 6 CR *P*. *aeruginosa* (0.8%), 5 CPE (0.6%), and 1 CR Acinetobacter spp. (0.1%); all of them were isolated from rectal swabs except two isolated from oropharyngeal swabs (Table 3). Moreover, five patients had an active infection with one of these microorganisms upon admission to the ICU (three by CR *P. aeruginosa*, and one CPE and EKP, respectively) (Table 3).

**TABLE 3:** Prevalence and incidence of colonization/infections by MDR-GNB in ICUs^*a*^^,b^

Hospital	ICU-admitted patients	Prevalence of colonization (%)	ICU patients admitted for at least 1 week	Patients colonized during the first admission week	Colonization incidence during the first week^*c*^	ICU patients admitted during 2 weeks	Patients colonized during the first two admission weeks	Colonization incidence during the first 2 weeks^*c*^	Number of patients infected at the time of admission (%)	Number patients infected during ICU stay (%)^*d*^
H1	156	3.2% (*n* = 5)	42	2	0.7 (*n* = 2)	12	2	1.2 (*n* = 2)	0	0
H2	87	10.3% (*n* = 9)	51	7	1.9 (*n* = 7)	24	14	4.2 (*n* = 14)	0	10 (11.5%)
H3	187	0.5% (*n* = 1)	50	1	0.3 (*n* = 1)	26	2	0.5 (*n* = 2)	0	1 (0.5%)
H4	76	1.3% (*n* = 1)	25	1	0.6 (*n* = 1)	9	1	0.7 (*n* = 1)	0	3 (3.9%)
H5	33	3% (*n* = 1)	11	0	0	6	0	0	0	1 (3%)
H6	142	3.5% (*n* = 5)	49	0	0	20	0	0	2 (1.4%)	0
H7	86	1.2% (*n* = 1)	53	0	0	24	3	0.9 (*n* = 3)	3 (3.5%)	1 (1.1%)
Total	**767**	**2.9% (*n* = 23**)	**281**	**11**	**0. 6 (*n* = 11)**	**121**	**22**	**1.3 (*n* = 22)**	**5 (0.7%)**	**16 (2%)**

	Colonizations upon ICU admission				Colonizations during the first 2 weeks of ICU stay			Infections during the period of study^*d*^		
Hospital	CPE^*e*^	ESBL-*Kpn*	CR *Pae*	CR *Aba*	CPE	ESBL-*Kpn*	CR *Pae*	CPE	ESBL-*Kpn*	CR *Pae*
H1	2 (Kpn)	3	0	0	0	0	2	0	0	0
H2	2 (Kox; Eh)	3	4	0	6 [Eh (4); Cit (2)]	1	7	4 (Eh)	0	6
H3	0	1	0	0	0	0	2	0	0	1
H4	0	0	0	1	0	1	0	0	2	1
H5	0	1	0	0	0	0	0	0	1	0
H6	1 (Kpn)	3	1	0	0	0	0	1 (Kpn)	1	0
H7	0	0	1	0	0	0	3	0	0	4
Total	**5**	**11**	**6**	**1**	**6**	**2**	**14**	**5**	**4**	**12**

^*a*^ Numbers of colonized and infected patients at ICU admission and density of incidence during the first and second week of admission.

^*b*^ Microorganisms were detected upon ICU admission and during the first week and the second week of ICU stay.

^*c*^ Incidence: (number of patients colonized during the first/second week of ICU stay/total of ICU patient-days in first/second week) × 100.

^*d*^ Up to a maximum of 60 days of stay in the ICU.

^*e*^ CPE, carbapenemase-producing Enterobacterales; CR, carbapenem-resistant; Kpn, *Klebsiella pneumoniae*; Pae, *Pseudomonas aeruginosa*; Aba, *Acinetobacter baumannii*; Kox, *Klebsiella oxytoca;* Eh, *Enterobacter hormaechei*; Eco, *Escherichia coli*; Cit, *Citrobacter* spp.

Among the 281 ICU patients admitted for at least 1 week, 11 acquired MDR-GNB colonization during the first week (overall incidence of 0.6 [11/1,967] cases per 100 ICU-admitted patients-day [interhospital range: 0–1.9]) (Table 3). The colonizing bacteria acquired were CR *P. aeruginosa* (eight patients), EKP (two patients), and CPE (one patient).

Among the 121 patients admitted to the ICU for at least 2 weeks, 22 were colonized by MDR-GNB in that period (overall incidence of 1.3 [22/1,694] cases per 100 ICU-admitted patients-day [interhospital range: 0–4.2]). Of them, 11 cases had already been detected in the first week of admission (Table 3). Colonization by CR *P. aeruginosa* was observed in 14 patients, CPE in 6, and EKP in 2 (Table 3).

Globally, MDR-GNB colonization was detected in 45 patients (the remaining up to 50 were admitted with active MDR infection without detectable colonization); 34 were identified in rectal samples, 4 in oropharyngeal samples, and 7 in both (Tables S1 and S2).

### Infections by MDR-GNB

Twenty-one patients had an infection by some of these bacteria during the study period (21/767; overall infection prevalence: 2.7%), five of them had an active infection at the time of admission and 16 acquired the infection during the ICU stay (six beyond the second week and up to day 60 of admission) (Table 4; Table S1). Twelve were CR *P. aeruginosa* (12/21; 57.1%), five CPE (5/21; 23.8%), and four EKP (4/21; 19%) (Table 3). In 16 (76.2%) of the patients who suffered an infection, colonization by the same bacteria was detected in at least one of the screenings carried out during their ICU admission (4/21; 19%) or ICU stay (12/21; 57.1%) (detailed in section Intrapatient and Interpatient Isolates In-depth Characterization; Table 4).

**TABLE 4:** Infections caused by the MDR-GNB under surveillance in this study upon admission and during the stay in the ICU (up to 60 days)

Time of diagnosis of infection	Type of infection (n)	Bacterial species/resistance indicator	Colonization
ICU admission	Primary bacteremia (2)	*K. pneumoniae*/ESBL and VIM-1	Yes
	Secondary bacteremia (1)	CR *P. aeruginosa*	Yes
	Urinary tract infection (1)	CR *P. aeruginosa*	Yes
	Respiratory tract infection (1)	CR *P. aeruginosa*	No
During the ICU stay (up to 60 days of stay)	Respiratory tract infection (1)	CR *P. aeruginosa*	Yes
	Respiratory tract/urinary tract infections (1)	*K. pneumoniae*/ESBL	Yes
	Respiratory tract (1)	CR *P. aeruginosa*	Yes
	Respiratory tract infection (2); urinary tract infection (1)	*K. pneumoniae*/ESBL (2); CR *P. aeruginosa* (1)	Yes (1); no (2)
	Respiratory tract infection (7); primary bacteremia (1); others (2)	CR *P. aeruginosa* (6); *E. hormaechei*/VIM-1(4)	Yes (8); no (2)

The infections due to MDR-GNB during the study were respiratory tract infections (12/21; 57.1%), bacteraemia (4/21; 19%), urinary tract infections (3/21; 14.2%), and other (2/21; 9.5%) (Table 4).

### Antibiotic susceptibility, STs, and resistance genes detected in MDR enterobacterales

A total of 45 Enterobacterales isolates from 24 patients were available for further microbiological and WGS analysis (Table S1). *K. pneumoniae* (*n* = 30) was detected in 16 different patients across six out of seven participating ICUs; *E. hormaechei* (*n* = 8) was found in 5 patients from the same ICU (Table S1).

Only the first isolate per species/resistance indicator for each patient was included for the analysis of resistance genes and antibiotic susceptibility (*n* = 26) (Tables 5 and 6). Among *K. pneumoniae* with ESBL as a unique resistance indicator (*n* = 13), CTX-M-15 producers predominated (*n* = 11; 84.6%). A carbapenemase gene was detected in the 13 remaining isolates, with *bla*_VIM-1_ being the most frequent (*n* = 12, 92.3%). Other VIM-1-producing Enterobacterales species are shown in Table 5; it should be noted that seven (53.8%) of the 12 VIM-1-producing Enterobacterales isolates co-produced an ESBL, mainly CTX-M-9 (5/7; 71.4%).

**TABLE 5:** Bacterial species distribution, resistance mechanisms, and main frequent STs detected in ^*a*^ICU patients colonized/infected by MDR-GNB

Bacteria and resistance indicator	Species	Total isolates (n)	Representative isolates (n)^*b*^	Main STs^*c*^	Resistance mechanisms
CPE and ESBL-producing *Klebsiella pneumoniae*	*K. pneumoniae*	30	16 (15 colonizations/1 infections)	ST307 (*n* = 4); ST15 (*n* = 3); ST584 (*n* = 2)	*bla*_CTX-M-15_ (*n* = 11); *bla*_CTX-M-209_+*bla*_SHV-27_ (*n* = 1); *bla*_VIM-1_(*n* = 1); *bla*_SHV-12_ (*n* = 1); *bla*_VIM-1_+*bla*_OXA-48_/*bla*_CTXM-15_ (*n* = 2)
	*K. oxytoca*	3	2 (colonizations)	ST46 (*n* = 1); ST86 (*n* = 1)	*bla*_CTX-M-9/_ *bla*_VIM-1_ (*n* = 1); *bla*_VIM-1_(*n* = 1)
	*E. hormaechei*	9	5 (colonizations)	ST133 (*n* = 3); ST78 (*n* = 2)	*bla*_VIM-1_/*bla*_CTXM-9_ (*n* = 4); *bla*_VIM-1_ (*n* = 1)
	*C. freundii*	1	1 (colonization)	ST98	*bla*_VIM-1_
	*C. amalonatycus*	1	1 (colonization)	ST987	*bla*_VIM-1_/*bla*_CTX-M-9_
	*E. coli*	1	1 (colonization)	ST410	*bla*_OXA-48_/*bla*_CTX-M-15_
CR *P. aeruginosa*	*P. aeruginosa*	37	20 (17 colonization/3 infections)	ST175 (*n* = 4); ST235 (*n* = 4); ST179 (*n* = 2); ST253 (*n* = 2)	OprD deficiency (*n* = 20); mutations in MexAB-OprM regulators (mexR *n* = 4, nalD *n* = 5)
CR *A. baumannii*	*A. baumanni*	1	1 (colonization)	ST3	*bla*_OXA-24_
	Total	83	47		

^*a*^ Only in 45 of the 50 patients colonized/infected by MDR-GNB isolates were available for molecular study.

^*b*^ First isolates per bacteria/resistance indicator for each patient selected for further microbiological and WGS analysis; three patients had two different bacteria/resistance indicators.

^*c*^ For *K. pneumoniae* and *P. aeruginosa,* only STs with more than one isolate are detailed. CPE, carbapenemase-producing *Enterobacterales*; CR, carbapenem resistant.

*K. pneumoniae* isolates (*n* = 16) were grouped into 10 STs (Table 5; Table S1). The most prevalent, ST307, was detected in four patients (25%, all CTX-M-15 producers), followed by ST15 detected in three patients (18.8%, two OXA-48+VIM-1 producers and one SHV-12 producer), and ST584 detected in two patients (12.5%, both CTX-M-15 producers). Likewise, the five representative *E. hormaechei* isolates belonged to ST133 (*n* = 3, all CTX-M-9 plus VIM-1 producers) and ST78 (*n* = 2) (Table 5; Table S1).

As shown in Table 6, all CPE showed resistance to at least one carbapenem (84.6%, 38.5%, and 7.7% of resistance to ertapenem, imipenem, and meropenem, respectively). Meropenem/vaborbactam and cefiderocol were active against 100% of Enterobacterales isolates. Susceptibility to meropenem/vaborbactam and imipenem/relebactam in VIM-1 isolates was justified by the low MICs, below the breakpoints, of meropenem and imipenem, and not by the activity of the inhibitors on VIM-1.

**TABLE 6:** Antibiotic susceptibility of the first 46 isolates per species/resistance indicator per patient collected in this study, determined by the microdilution method according to EUCAST clinical breakpoints

	Enterobacterales (*N* = 26)^*b*^	CPE (*N* = 13)	ESBL-producing *Klebsiella pneumoniae* (*N* = 13)	CR *P. aeruginosa* (*N* = 20)
Antibiotics	Susceptibility (%)			Susceptibility (%)
Amoxicillin/clavulanic	19.2	0	38.5	ND^*c*^
Piperacillin/tazobactam	30.7	0	61.5	25
Cefotaxime	0	0	0	ND
Ceftazidime	0	0	0	45
Cefepime	23	15.4	30.8	55
Cefiderocol^*a*^	100	100	100	95
Ceftazidime/avibactam	46.2	7.7	84.7	100
Ceftolozane/tazobactam	42.3	0	84.6	100
Aztreonam	34.6	69.2	0	55
Ertapenem	57.7	15.4	100	ND
Meropenem	96.1	92.3	100	45
Imipenem	80.8	61.5	100	0
Meropenem/vaborbactam	100	100	100	ND
Imipenem/relebactam^*a*^	76.9	53.8	100	90
Gentamicin	42.3	23	61.5	ND
Tobramycin	26.9	0	53.8	85
Amikacin	96.2	100	92.3	85
Ciprofloxacin	19.2	0	38.5	20
Tigecycline	53.8	38.5	69.2	ND
Eravacycline	65.4	53.8	76.9	ND
Trimethoprim/sulfamethoxazole	7.7	7.7	7.7	ND
Fosfomycin	76.9	69.2	84.6	ND
Colistin	100	100	100	100

^*a*^ Antimicrobial susceptibility was determined by the broth microdilution method following the procedure recommended by EUCAST (20).

^*b*^ Including ESBL-producing *Klebsiella pneumoniae* and carbapenemase-producing Enterobacterales.

^*c*^ ND, not determined.

The most active non-beta-lactam antibiotics against Enterobacterales were colistin and amikacin with 100% and 96.2% of susceptibility, respectively (Table 6).

The non-β-lactamase ARGs detected in at least 60% of the Enterobacterales isolates were as follows: the sulfonamide-resistant-encoding genes *sul* (*n* = 25/26, 96.2%); the quinolone-resistant-encoding *qnr* (*n* = 24, 92.3%); the trimethoprim-resistant-encoding *dfr*A (*n* = 17/26, 65.4%); the aminoglycoside-resistant-encoding *aac(6′)-Ib-cr* (*n* = 17, 65.4%); and the chloramphenicol-resistant-encoding cat genes (*n* = 16, 61.5%) (Table S1).

### Antibiotic susceptibility, STs, and resistome analyses in CR *P. aeruginosa* and *Acinetobacter* spp*.*

A total of 37 *P. aeruginosa* isolates from 20 different patients and one *A. baumannii* was available for further analysis (Table S2). As for the Enterobacterales, only the first isolate per resistance indicator from each patient was included for analyzing antibiotic susceptibility and resistance mechanisms (*n* = 20).

As shown in Table 6, all *P. aeruginosa* isolates were resistant to imipenem, with 55% also resistant to meropenem and 40% susceptible at increased exposure. Susceptibility rates for ceftolozane/tazobactam, ceftazidime/avibactam, imipenem/relebactam, and cefiderocol were ≥90%. Among non-beta-lactam antibiotics, colistin was the most active one; all isolates were susceptible, followed by amikacin and tobramycin, with 15% of resistant isolates.

Selected *P. aeruginosa* isolates (*n* = 20) grouped into 12 STs, being high-risk clones ST175 and ST235 the most prevalent ones, both detected in four different patients (Table 5; Table S2).

None of the 20 CR *P. aeruginosa* isolates produced a carbapenemase. Instead, imipenem resistance was attributed to chromosomal events leading to OprD deficiency (Table 6; Table S2). In addition, six isolates harbored mutations in *mexR* (*n* = 4) and *nalD* (*n* = 2), both regulators of the MexAB-OprM efflux pump. A well-known gain-of-function mutation (*ftsI*-F533L) within the gene coding for the penicillin-binding-protein 3 was detected in the two isolates exhibiting the highest MICs for meropenem (>64 mg/L).

Half of the isolates carried non-β-lactamase acquired resistance genes (Table S2). Aminoglycoside-modifying enzymes were found in high-risk clones ST175, ST179, and ST235 isolates, and genes conferring resistance to other antibiotics were also detected in the two ST179 isolates, both harboring a quinolone resistance pentapeptide repeat protein (*qnrVC1*) and a dihydrofolate reductase (*dfrA14)* (Table S2).

Finally, the single CR *A. baumanni* isolate detected during the study period belongs to ST3, was only susceptible to colistin, and harbored *bla*_OXA-24_ (Table 5).

### Intrapatient and interpatient isolates in-depth characterization

There were 22 patients in whom more than one MDR-GNB isolate was detected. In three of these patients, two different bacterial species/resistance indicators were identified: (i) patient H2-048, who acquired a co-colonization by a VIM-1-producing *C. freundii* and a CR *P. aeruginosa*; (ii) patient H2-055, colonized by a CTX-M-15-producing *K. pneumoniae* at ICU admission and acquired a CTX-M-15 plus OXA-48-producing *E. coli* later; and (iii) patient H2-068, who suffered double colonization during admission with a VIM-1-producing *K. oxytoca* (rectal swab) and a VIM-1-producing *E. hormaechei* (oropharyngeal and rectal swabs). In all other patients, a unique bacterial species/resistance indicator was detected in sequential samples (Tables S1 and S2).

Of note, 10 patients from whom we have sequential isolates of colonization and infection (seven colonized by a CR *P. aeruginosa*, two by *K. pneumoniae,* and one by *E. hormaechei*) suffered an infection during their ICU stay due to the same bacteria that colonized them (Tables S1 and S2).

Phylogenetic trees were constructed using the gene-by-gene approach and allelic distances from cgMLST for all available *K. pneumoniae* isolates (*n* = 30), *E. hormaechei* (*n* = 9), and *P. aeruginosa* (*n* = 37).

**Fig 2:**
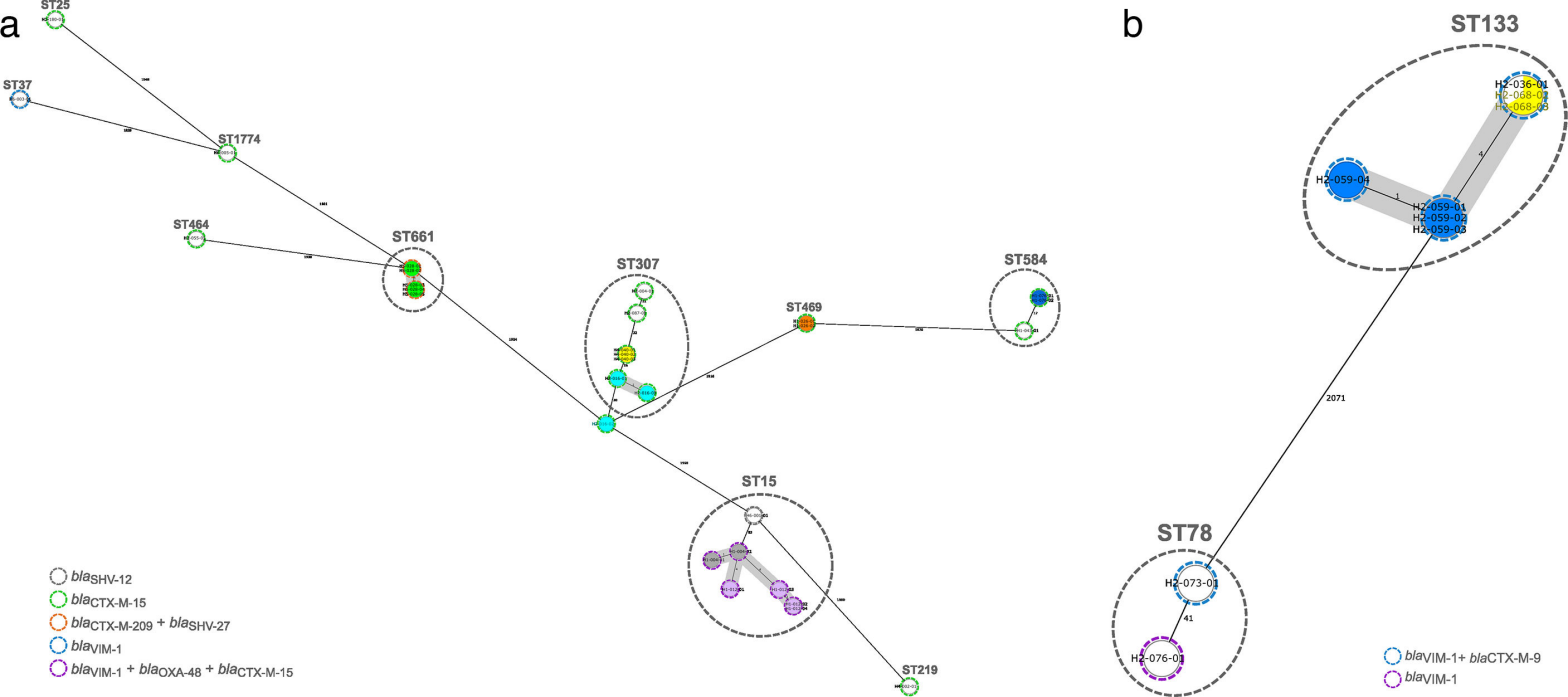
Phylogenetic tree of the enterobacterales population. (**a**) Population structure of the K. *pneumoniae* isolates detected in this study. The distances shown in the minimum spanning tree are based on the cgMLST scheme of 2,538 genes (Ridom SeqSphere+). (**b**) Population structure of the *E. hormaechei* isolates detected in this study. The distances shown in the minimum spanning tree are based on the cgMLST scheme of 2,123 target genes and 1,798 accessory genes. Fill circles of the same color that correspond to sequential strains from the same patient. Fill white circles that correspond to only one isolate from a patient. The color of the dashed line in circles indicates the ESBL and carbapenemase types. Gray ovals represent clusters, and the shading indicates a threshold of ≤4 alleles of difference between the isolates.

Figure 2a shows the genetic relationship of *K. pneumoniae* isolates in relation to their main resistance mechanisms and patients. Seven genetic groups (≤4 alleles of difference) were characterized as follows (Fig. 2a): Group (1) five CTX-M-209+SHV-27/ST661 isolates; Groups (2 and 3) two groups of three CTX-M-15/ST307 isolates; Group (4) two CTX-M-15/ST469; Group (5) two CTX-M-15/ST584 isolates; and Groups (6 and 7) two groups of four and three OXA-48+VIM-1+CTX-M-15/ST15 isolates, respectively. All genetic groups were formed by isolates from the same patient, except OXA-48+VIM-1+CTX-M-15/ST15 isolates, which came from two different patients admitted at the same ICU (Fig. 2a; Table S1).

Figure 2b shows the cgMLST analysis of nine *E. hormaechei*, with seven ST133 isolates producing VIM-1 and CTX-M-9 forming a single group with ≤4 alleles of difference (average of 2.5 allelic differences; range: 0–5) obtained from three patients admitted at the same ICU (Table S1).

As shown, the majority of the Enterobacterales sequential isolates (*K. pneumoniae* and *E. hormaechei)* from the same patient (in eight of the nine patients) belonged to the same ST and were identical or very similar by cgMLST (≤4 alleles of difference) (Fig. 2a and b). Just one exception to this was identified suggesting probable different colonization events; this case was formed by four sequential OXA-48+VIM-1+CTX-M-15/ST15 *K. pneumoniae* isolates, one of which shows allelic differences of 8–9 alleles in pairwise comparisons (Fig. 2a). The range of allelic distance in paired comparisons in isolates from the same patient was 1–9 in *K. pneumoniae* (Fig. 2a). Moreover, the ARGs of the sequential Enterobacterales isolates did not significantly change between isolates of the same ST and from the same patient (*n* = 9 patients) (Table S1)*.*

**Fig 3:**
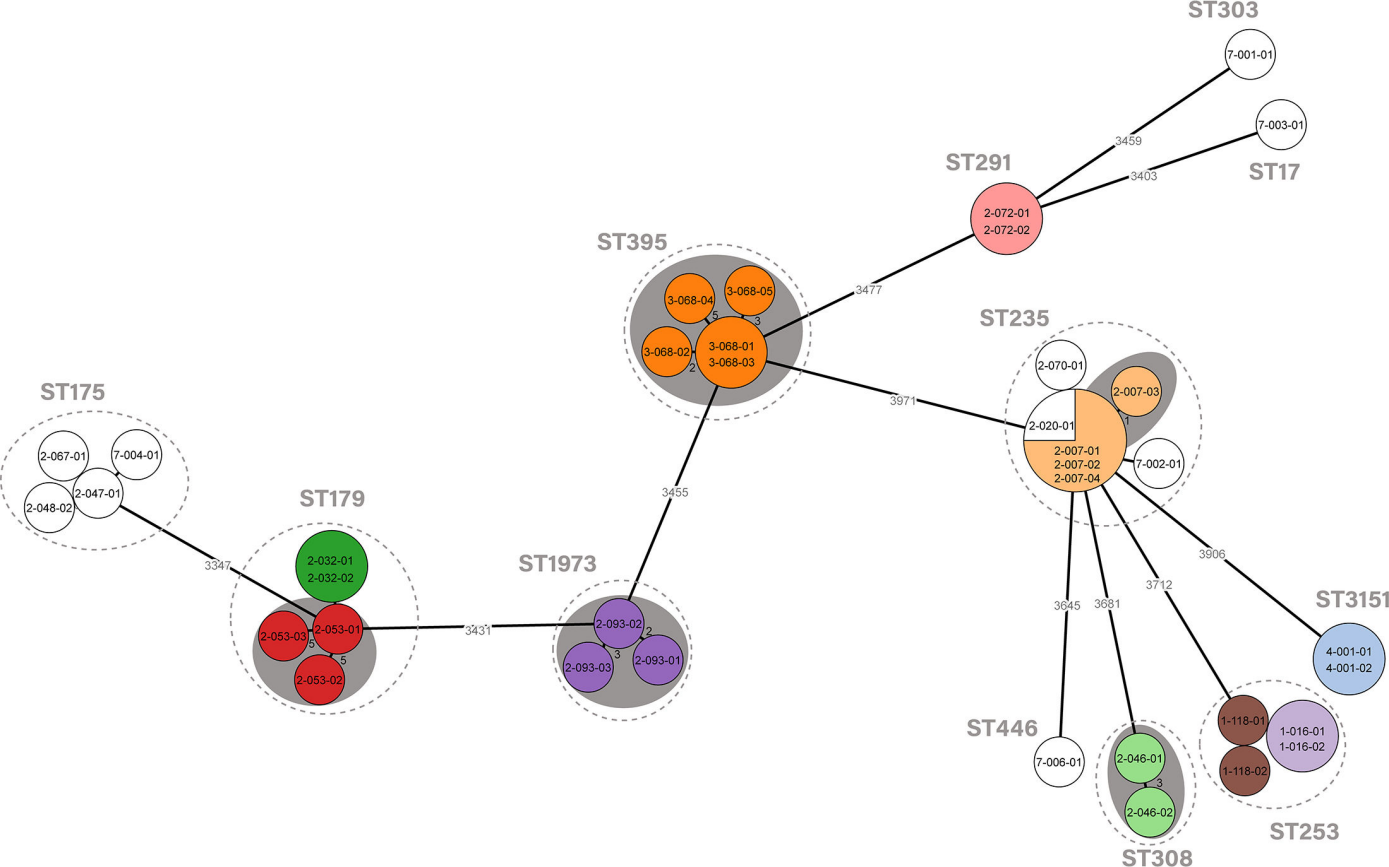
Phylogenetic tree of the *P. aeruginosa* population detected in this study. The distances shown in the minimum spanning tree are based on the cgMLST scheme of 4,201 genes (chewBBACA). Circles of the same color correspond to sequential strains from the same patient. Fill circles of the same color that correspond to sequential strains from the same patient. Fill white circles that correspond to only one isolate from a patient. Gray ovals represent clusters, and the shading indicates a threshold of ≤6 alleles of difference between the isolates.

The genetic relatedness among *P. aeruginosa* isolates is represented in Fig. 3. All isolates obtained from the same patient grouped (≤6 alleles of difference) except for patient H1-118, for which seven allelic differences were documented between its two isolates. All genetic groups were formed by isolates from the same patient, with the single exception of ST235 isolates that included two patients (H2-007 and H2-020, range of allelic differences 0–1).

Moreover, sequential CR *P. aeruginosa* isolates obtained from the same patient also showed an identical acquired and mutational resistome with the only exception of patient H2-046, for which a different mutation in the AmpC regulator *ampD* was documented between the colonizing and infecting isolate.

Finally, antibiotic susceptibility in sequential isolates (same species and ST) from a single patient was generally very similar. In none of these strains, exogenous acquisition of additional resistance genes was observed, so they are probably due to changes in the expression or activation of chromosomal mechanisms.

## DISCUSSION

Surveillance of ARB is essential for optimizing therapy and controlling resistance spread. Carrier screening, particularly in ICUs, is a key outbreak management tool recommended by the ECDC (1, 5, 32). “Zero Resistance” is a Spanish program that enhances surveillance, infection prevention, and antimicrobial stewardship to curb resistance, especially in high-risk healthcare settings (33).

This comprehensive study integrates incidence/prevalence, clinical, microbiological, and genomic data to draw the current picture of MDR-GNB in Spanish ICUs. Its main strengths are the comprehensive multidisciplinary approach to the health problem, as well as the participation of seven ICUs of large university hospitals from different Spanish regions. The main limitation is the fact that each participant ICU presents a different bacterial ecosystem and different infection prevention and control strategies, as well as cleaning procedures that could influence the individual hospital variations observed in the prevalence and incidence of ICU-acquired MDR-GNB.

Notably, both prevalence of MDR-GNB upon ICU admission and incidence of colonization detected for 2 weeks were relatively low in this study, although important individual hospital variations were observed. It should be noted that a previous trend toward the increase of PCE prevalence in Spain had been communicated (4,500 cases of PCE were voluntarily reported in 2022 [34]), which could indirectly indicate the improvement of good practices in detection and control of MDR-GNB in Spain.

Although oropharyngeal and rectal swab results were combined to calculate the total number of colonizations, intestinal colonization was much more frequent.

There are few integrated studies on the incidence of high-priority MDR-GNB carriers in ICUs, as most addressed individual pathogens. In a meta-analysis of 13 studies with 15,045 ICU patients, the acquisition rate of digestive tract colonization by ESBL-producing Enterobacterales during ICU stay was 7%, and it varied from 3% to 4% in the Americas and Europe to 21% in the Western Pacific region (35). In Spain, a previous similar study on CPE carried out in 2014 showed a prevalence of 0.61% on admittance, similar to the one in this study, considering only CPE (16); although the general epidemiological status of CPE in Spain had evolved significantly between that study and the one communicated here (36).

Additionally, this study showed that previous colonization by MDR-GNB is one of the main risk factors for infection by these pathogens in the ICU, since up to 35% of colonized patients were infected by the same bacteria, while only 2.7% of the total number of patients admitted during the study period were infected by these pathogens (35, 37). A recent meta-analysis about CR *K. pneumoniae* showed an incidence of infections after colonization of 23.2% (37). In another study on CP *Enterobacterales*, the risk of bacteremia among CPE was higher in ICU and oncology wards than in other settings (38). Additionally, recent works on the evolution of the microbiome in ICU patients have correlated the increasing presence of multi-resistant pathogenic bacteria in the patient’s microbiome with an increased infection risk (39).

Some of the most internationally recognized high-risk clones were detected in Spanish ICUs, such as ST15 and ST307 in *K. pneumoniae* or ST175, ST179, and ST235 in *P. aeruginosa. K. pneumoniae* ST307 has been described as one of the most rapidly emerging *K. pneumoniae* high-risk clones producing ESBLs and carbapenemases (36, 40). *P. aeruginosa* high-risk clone ST175 has been frequently detected in Spanish hospitals, with being MDR phenotype linked to mutation-driven resistance mechanisms. However, in recent years, ST179 and ST235 high-risk clones, which frequently harbor carbapenemase-encoding genes, have been increasingly detected in our country (41). The relevance of these high-risk clones in the spread of antimicrobial resistance has been raised in the last years (42), making it necessary to integrate genomic sequencing into surveillance for their accurate detection (19).

The most recently introduced beta-lactam/beta-lactamase inhibitors showed over 95% *in vitro* activity toward all isolates. However, for enterobacterales, this rate dropped for imipenem/relebactam (showed over 75% *in vitro* activity) because most of the isolates resistant to it were VIM-1 producers, in agreement with the weak activity of this antibiotic combination against class B carbapenemases (43). As previously described (44), cefiderocol showed good activity *in vitro* against all isolates, irrespective of the carbapenemase types. All Enterobacterales isolates resistant to ceftazidime/avibactam (12/26; 46.1%) were VIM-1, OXA-48, or VIM-1/OXA-48 producers. KPC variants associated with a susceptibility decrease to ceftazidime/avibactam were not detected in this study (45, 46).

Taken together, our data show that the most frequent resistance mechanisms and clones detected are the same ones that circulate in Spain. Therefore, the bacterial ecosystem of each ICU mainly reflects the epidemiology of the hospital to which it belongs and its geographic region. So, monitoring the local ICUs epidemiology of resistance to multiple antibiotics, including molecular surveillance of mechanisms and clones, should be a fundamental tool in the appropriate use of antimicrobials and in the design of stewardship and infection control programs. In addition, this study reinforces the idea that one of the main risk factors for MDR-GNB infection in ICUs is previous colonization. Since CR *P. aeruginosa* was the most frequent bacterium identified in colonizations and infections, it should be included in the routine hospital colonization cultures.

## Data Availability

Bacterial genome data (raw Illumina reads) are publicly available in the European Nucleotide Archive (ENA): PRJEB78527 (Enterobacterales) and PRJEB88879 (*P. aeruginosa* and *Acinetobacte*r spp).

## SUPPLEMENTAL MATERIAL

Table S1 spectrum.02987-25-s0001.xlsx Molecular, microbiological and clinical data of the 45 *enterobacterales* isolates included in the study.

Table S2 spectrum.02987-25-s0002.xlsx Molecular, microbiological and clinical data of the 37 *Pseudomonas aeruginosa* isolates included in the study.

ASM does not own the copyrights to Supplemental Material that may be linked to, or accessed through, an article. The authors have granted ASM a non-exclusive, world-wide license to publish the Supplemental Material files. Please contact the corresponding author directly for reuse.
